# Brief Commentary: Why We Need More Equitable Human Resources for Health to Manage the Covid-19 Pandemic

**DOI:** 10.3389/fpubh.2020.573742

**Published:** 2020-11-02

**Authors:** Charlotte Scheerens, Jan De Maeseneer, Tobias Haeusermann, Milena Santric Milicevic

**Affiliations:** ^1^Institute for Lung Health, Beth Israel Deaconess Medical Center, Harvard Medical School, Boston, MA, United States; ^2^Department of Public Health and Primary Care, Ghent University, Ghent, Belgium; ^3^Department of Geriatrics, Faculty of Medicine, University of California, San Francisco, San Francisco, CA, United States; ^4^Department of Sociology, University of Cambridge, Cambridge, United Kingdom; ^5^Faculty of Medicine, University of Belgrade, Belgrade, Serbia

**Keywords:** human resources for health, equity, COVID-19, health governance, data monitoring, social determinants of health, health care coordination

With this brief commentary we urge human resources for health (HRH), i.e., all people engaged in actions whose primary intent is to enhance health^1^, to be more equitable if we want to minimize the disproportionate impact of covid-19 in regions with unfair health systems such as the United States (U.S.). As it is so often the case in societies with inequitable access to, and inappropriate distribution of public health resources, crises like the outbreak of severe acute respiratory syndrome coronavirus 2 (SARS-CoV-2) hits disadvantaged groups the hardest. We see that occurring not only in low-income countries in Africa and South-America with constrained health systems, but also in Europe (1) and the U.S. (2) in particular. While the virus does not discriminate, inappropriate health systems certainly do. Race, ethnicity, and class disparities, aggravated by decade-long exclusion from high-quality health workers, health care services and comprehensive insurance, are reflected both in the virus's morbidity and mortality rates (3–5) as well as the fallout from the unfolding socioeconomic impact across communities (2).

Indeed, entrenched health inequity [i.e., avoidable and remediable consequences of structural health injustices (6)] between groups of people presented a global health threat long before COVID-19 (7), yet the pandemic has significantly compounded the impact of inappropriate medical care systems, capacities of health workers and technologies on health disparities (8). Therefore, if we are to advance health equity (9) and population health (10), not only as a public health issue, but also as an urgent matter of justice in the health systems' responsiveness (7), more emphasis on social justice and equity in the decision making process of health system's resources generation and allocation is needed (11). This is particularly true for equity in HRH (11).

We suggest that the path to improving HRH equity entails inclusive governance that creates a fair and accessible health care system with an appropriate distribution of competent and motivated health workers. The latter needs to be fit for purpose and practice in their respective context, and appropriately meet the varying needs of all communities they serve, especially the most underserved. We argue that HRH equity is the foundation for accelerating universal health coverage (UHC) (11) which may ultimately lead toward attaining Sustainable Development Goals such as Health and Well-being for all (Goal 3) (12). Here, we highlight how pre-existing health and HRH inequities rendered appropriate responses to the pandemic more challenging. If we plan to emerge from this crisis in a better position, we need a guiding framework on HRH equity that entails indicators with a specific focus on HRH inequities.

## A Case In Point of Inequitable HRH Governance

In some U.S. states and federally, mitigating HRH inequities was not a governance priority (13). Since the covid-19 pandemic struck in early 2020, health care providers and patients in all countries had to act fast to alleviate the cumulative impacts of the outbreak (14). The nursing workforce in the U.S. —the largest health professionals group—was rapidly depleted as demands for frontline health workers spiked (15), especially in disadvantaged neighborhoods. Even pre-covid-19, the nursing profession was at an estimated shortage of one million workers (15), chiefly due to a lack of nursing faculty, high turnover, and inequitable distribution of the workforce (16). The actual budget (17) further exacerbated these circumstances by the state's failing to provide sufficient health funding while simultaneously cutting funds for nursing workforce development programs by 64 percent. According to the American Nurses Association, these cuts “essentially dismantled programs that recruit, train, and educate nurses for practice in rural and medically underserved communities” (17). As a result, current health workforce measures include overtime work, delayed annual leave (18), and reactivation of retired health workers throughout the country (19). Moreover, nurse staffing agencies in the U.S. resorted to offering unprecedented incentives for those willing to enter hot zones, including up to $10,000 a week in crisis pay, relocation bonuses, tax-free housing and food (15). Whereas conforming to these extreme measures almost elevated the health workforce to the status of nationwide superstars in the U.S. and worldwide, these measures painfully show that the way out of covid-19 must lead to a path toward equitable HRH.

Furthermore, a misbalance in care access also occurs when the health workforce is inappropriately organized. Health systems lacking a community-oriented (primary) health care system that integrates with public health services face difficulties in meeting individuals' needs while staying-in-shelter as part of an appropriate health response to the virus. In support to the fieldwork of public health specialists, primary health care professionals keep oversight of the differing needs of people at home (20). Meanwhile, clinical specialists are crucial to attend to patients during hospitalization or emergency room visits. Hence, the equitable scaling up of multi-, trans- and inter-professional teams to work in clinics and health centers in the community (such as public health specialists, family practitioners, nurses, clinical specialists, community workers and social workers), will contribute to the most effective difference in the context of health and well-being for all (21, 22).

Some centralized and coordinated strategies come with various advantages, particularly in view of the dangers posed by public health emergencies that transcend borders and local sovereignties (23). First, creating a task force involving all ministries along with all regional and municipal governments helps reach a greater percentage of the population. This would facilitate the containment of a virus. Second, a joined-up strategy and an all-government approach helps extending benefit packages and improve equity of care. If the goal of the country is to reduce health inequities, equitable access to qualified and licensed health workforce ought to be enshrined as a human right by switching toward UHC. Instead of only (re-)acting in crises while cutting budgets in the interim, there is an urgent need for a country's leadership to invest more and continually in HRH equity, as it enhances effective, accessible, equitable and affordable health care for all, and empowers the government to cut down costs of a re-organized and integrated health care system.

## The Way Forward to HRH Equity

We believe that reaching equity in HRH requires close engagement with stakeholders across governments, sectors and communities. It should entail comprehensive and multi-sectoral action at all levels, with implementation and development of accountability and sustainability mechanisms to manage the health workforces' production, stock, skill-mix, distribution, accessibility, productivity and quality (11). Stakeholders must have credible systems to regularly assess evolving population needs, monitor progress on delivery using HRH equity-related indicators, and more importantly, harness the data and findings to evaluate and adjust HRH policies, programs and action plans.

The best health leadership and inclusive governance are only possible if governments start tracking and tracing HRH equity solutions. This applies to both monitoring health progress in the current and post-pandemic situation, as well as addressing long-term HRH inequities. Consequently, public health officials and services partners need granulated, reliable, relevant, and timely data on HRH, enabling a comprehensive overview of the range of health workforce from health sciences, medical and nursing school enrollment numbers to geographical health workforce spread and its impact on social determinants of health. To collect such comprehensive data, tools are needed to help prioritize scarce investigative resources (24). The WHO's National Health Workforce Accounts (NHWA) provides an example that can increase and strengthen such HRH data and ultimately provide the knowledge necessary for countries to improve data availability, quality, standardization and usage (25). While the NHWA and most other tools focus on the health workforce in general, no tools or unified set of indicators specifically focus on HRH inequities. This is partly due to context-bound priorities, which can differ within and between countries, but also due to certain difficulties in defining, measuring, detecting and preventing HRH inequities buried in the public and private policy system. It would therefore be extremely useful if international bodies develop a guiding HRH equity framework, with key concepts, definitions and indicators to support health systems development in the right direction.

While this global pandemic imposed enormous human and economic costs, it equally exposed core issues of health workforce inequity inherent in many current health systems. Health leaders who go forward ought to envision health systems that translate the lessons learned on health equality and social justice into new forms of knowledge on HRH equities. With other challenges of enormous proportions such as migration and climate change around the corner, the current HRH policies and governance related to their availability, composition, deployment and work quality would need a drastic revision, upgrade, and investment if we want to guarantee dignified lives for all people worldwide.

## Author Contributions

CS: conceptualization and writing first draft. All authors: writing final draft.

## Conflict of Interest

The authors declare that the research was conducted in the absence of any commercial or financial relationships that could be construed as a potential conflict of interest.

## Footnotes

^1^https://www.who.int/healthinfo/statistics/toolkit_hss/EN_PDF_Toolkit_HSS_HumanResources_oct08.pdf?ua=1
